# Pro/antioxidant status and selenium, zinc and arsenic concentration in patients with bipolar disorder treated with lithium and valproic acid

**DOI:** 10.3389/fnmol.2024.1441575

**Published:** 2024-09-11

**Authors:** Joanna Rog, Łukasz Łobejko, Michalina Hordejuk, Wojciech Marciniak, Róża Derkacz, Adam Kiljańczyk, Milena Matuszczak, Jan Lubiński, Miłosz Nesterowicz, Małgorzata Żendzian-Piotrowska, Anna Zalewska, Mateusz Maciejczyk, Hanna Karakula-Juchnowicz

**Affiliations:** ^1^Laboratory of Human Metabolism Research, Department of Dietetics, Institute of Human Nutrition Sciences, Warsaw University of Life Sciences (WULS-SGGW), Warsaw, Poland; ^2^Mental Health Center at the Independent Public Healthcare in Leżajsk, Leżajsk, Poland; ^3^1st Department of Psychiatry, Psychotherapy and Early Intervention, Medical University of Lublin, Lublin, Poland; ^4^Department of Genetics and Pathology, International Hereditary Cancer Center, Pomeranian Medical University, Szczecin, Poland; ^5^Read-Gene, Grzepnica, Poland; ^6^Students’ Scientific Club “Biochemistry of Civilization Diseases” at the Department of Hygiene, Epidemiology and Ergonomics, Medical University of Bialystok, Bialystok, Poland; ^7^Department of Hygiene, Epidemiology and Ergonomics, Medical University of Bialystok, Bialystok, Poland; ^8^Independent Laboratory of Experimental Dentistry, Medical University of Bialystok, Bialystok, Poland

**Keywords:** oxidative stress, biomarkers, trace elements, arsenic, selenium, zinc, psychiatric disorders, nutritional psychiatry

## Abstract

Disturbances in pro/antioxidant balance emerge as a crucial element in bipolar disorder (BD). Some studies suggest that treatment effects on trace element concentration in BD. This study aimed to identify (a) the changes related to oxidative stress in BD and their relationship with trace elements engaged in pro/antioxidant homeostasis; (b) BD biomarkers using machine learning algorithm classification and regression tree (C&RT) analysis. 62 individuals with BD and 40 healthy individuals (HC) were included in the study. The concentration of pro/antioxidant state and concentration of selenium, zinc, arsenic in blood were assessed. We found a higher concentration of total antioxidant capacity, catalase, advanced oxidation protein products and a lower concentration of 4-hydroxynonenal (4-HNE), glutathione, glutathione peroxidase (GPx) in BD compared to HC. All examined trace elements were lower in the BD group compared to HC. A combination of two variables, 4-HNE (cut-off: ≤ 0.004 uM/mg protein) and GPx (cut-off: ≤ 0.485 U/mg protein), was the most promising markers for separating the BD from the HC. The area under the receiver operating characteristic curve values for C&RT was 90.5%. Disturbances in the pro/antioxidant state and concentration of trace elements of patients with BD may be a target for new therapeutic or diagnostic opportunity of BD biomarkers.

## Graphical Abstract

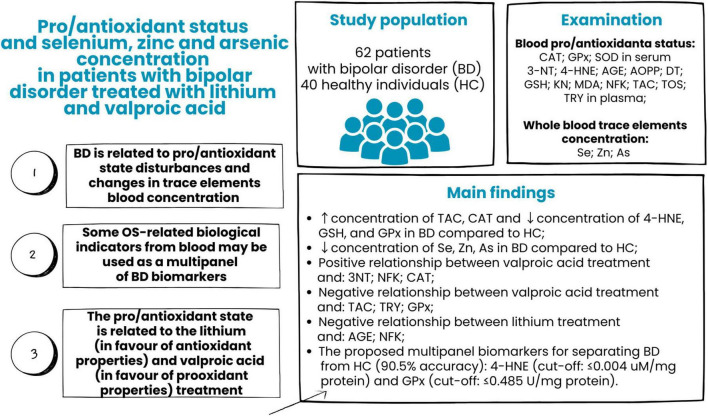

## 1 Introduction

Bipolar disorder (BD) is a chronic mental illness and is marked by an alternation between mania/hypomania and depression (elevated and decreased mood). BD is one of the ten leading causes of disability around the world. Pharmacological treatment is well-known, standardized and tolerated by patients, yet drug tolerance and therapy effectiveness vary among patients. It is worth noting that despite the existence of standard therapeutic protocols, individual differences in treatment responses and the occurrence of side effects pose a significant challenge. Additionally, incomplete response, relapse of mood episodes and suboptimal outcomes remain the pharmacotherapy a challenge (Jain and Mitra, 2024). Despite BD symptoms, most patients are not correctly diagnosed until 6 to 10 years after the clinical expression. Misdiagnosis and long-time delayed diagnosis are the results of an unrecognized transition from depression to elevated mood symptoms (hypomania or mania) (Mesbah et al., 2024). An incorrect diagnosis leads to untailored treatment strategies, progression and a worse course of the disease. A wide range of clinical manifestations often overlap between psychiatric disorders (Keck et al., 2008). Moreover, diagnosis is based on clinical interview and syndromic descriptive classification criteria and could be influenced by subjective biases of clinical practitioner or patient. The inclusion of more objective measures of mental health in clinical practice would avoid this bias.

Several studies employ biological markers as a potential tool to aid the clinical diagnosis of BD or treatment response (Grewal et al., 2023; Hu et al., 2023; Chen et al., 2024). Peripheral blood, as easily accessible and routinely examined, poses an attractive opportunity for early, more precise diagnosis and monitoring of patients’ health. Among the factors that are easily assessed in blood and could be engaged in the pathophysiology of BD, oxidative stress (OS) gains a lot of attention (Wartchow et al., 2023).

OS is an imbalance between antioxidant defenses and free radical production. OS leads to a disruption of redox signaling and control and/or molecular damage (Jomova et al., 2023). When free radicals are overproduced, a disruption of redox signaling controls and/or damages biological molecules, such as proteins, DNA, and lipids (Cecerska-Heryć et al., 2022). OS may interact with immune-inflammatory pathways and contribute to neurodegeneration (Teleanu et al., 2022). The negative impact of pro/antioxidant imbalance on the human body could be diminished by counteracting antioxidant enzymes: superoxide dismutase (SOD), glutathione peroxidase (GPx) or catalase (CAT). Lastly, more attention is paid to pro- and antioxidants delivered (intentionally or unintentionally) with food. Selenium (Se) is an important antioxidant and active micronutrient. Twenty-five selenoproteins (including SOD) in humans are involved in a wide variety of biological functions, at least. It has been shown that Se is involved in various biological processes related to mental disorders, such as neurotransmission, maintaining pro/antioxidant and pro/anti-inflammatory homeostasis (Bjørklund et al., 2022). The decreased blood levels of Se in individuals suffering from mental disorders, compared to healthy individuals, have been reported in some studies (Baj et al., 2020; Santos et al., 2022). It remains unclear whether Se status may be associated with the etiology of BD. However, the model studies find Se protective properties against diverse side effects related to lithium (Li) treatment (widely used in BD), including distribution in microelements homeostasis [including zinc (Zn), which is suggested to reduce or prevent depressive symptoms], changes in antioxidant enzymes blood levels and oxidant disturbances in the brain (Kiełczykowska et al., 2014, 2018; Kielczykowska et al., 2015). The negative impact of arsenic (As) on metabolic pathways modulated by Se has been shown. The authors suggest avoiding using Li in patients vulnerable to As exposure (f.e., by drinking water or living in an arsenic-contaminated area) because Li enhances the adverse effects caused by As (Bhardwaj et al., 2018).

Increasing efforts to identify biomarkers specific to BD have been made over the past few years. However, the clinical utility of the biomarkers proposed by the researcher still needs to be improved. The possibility of multiple assessments from one blood sample allows for a more complex evaluation of parameters reflecting disturbances in BD patients.

The aim of the study was to identify (a) the potential changes related to oxidative stress in BD patients and their relationship with trace elements engaged in pro/antioxidant homeostasis; (b) BD biomarkers using machine learning algorithms to improve diagnosis and treatment safety.

## 2 Materials and methods

### 2.1 Participants

The study was conducted according to the ethical principles of the Declaration of Helsinki (World Medical Association, 2013), and the protocol of the study was approved by the Ethics Committee of the Medical University of Lublin, Lublin, Poland (ID: KE-0254/201/2017). Before entering the study, all patients were informed about its procedure: the objectives and methods, anonymity, potential benefits and risks associated with participation, and volunteer participation by the same study physician. Informed consent was explained and signed in written for by the patient.

The group of 62 individuals (23 females and 39 males) with BD diagnosis was included in the study (BD group). BD group was inpatients recruited from the 1st Department of Psychiatry, Psychotherapy and Early Intervention of the Medical University of Lublin, the Independent Public Provincial Psychiatric Hospital in Radecznica, the Autonomous Public Healthcare in Leżajsk and outpatients from private medical practice psychiatrists from Lublin. The median age of patients was 37 years (18–65).

The following inclusion criteria to BD group were adopted: (1) obtaining informed written consent for participation in the study; (2) diagnosis of BD according to The International Classification of Diseases, 10th revision (ICD-10) criteria (CDC, 2024); (3) age between 18 and 65 years old; (4) treatment of Li and/or valproic acid (VA) within the therapeutic range of blood concentration and symptomatic remission during entry of the study. The exclusion criteria in the BD group were: (1) lack of informed written consent; (2) cancer diagnosis, neurological disease or organic brain dysfunction, inflammatory diseases in active phase or any other disease in an unstable phase; (3) any addiction (except nicotine and caffeine); (4) supplementation of Se and/or Zn; and (5) pregnancy or lactation. No restrictions on additional medication were established.

The control group (HC) comprises 40 healthy individuals (15 females and 25 males) whose age, gender, and body mass index (BMI) matched BD. The HC group consisted of staff, their relatives, and students from the hospitals mentioned above. The exclusion criteria for the HC group were the same as in the BD group, and additionally, the presence of any past or current axis I or II psychiatric disorder.

### 2.2 Data collection

#### 2.2.1 Biological assessment

##### 2.2.1.1 Blood collection

Venous blood (25 ml) was collected after 8-h fasting. To obtain the serum and plasma, the samples were centrifuged (at 2,000 × *g*, 20 min at room temperature), and equal aliquots of supernatants were taken to Eppendorf tubes (Eppendorf^®^), frozen and stored at −80°C before further analysis.

Whole blood samples were collected from patients, transported to the laboratory, and stored in plastic tubes at −80°C until analysis. Before thawing, the samples were centrifuged (at 4,500 × *g*, 15 min), and the supernatant was diluted 20-fold with 1% nitric acid solution (Suprapur^®^ HNO3; Merck, Germany). The following parameters were analyzed in the samples:

(a) Serum: catalase (CAT), glutathione peroxidase (GPx), superoxide dismutase (SOD);
(b) Plasma: 3-nitrotyrosine (3-NT), 4-hydroxynonenal (4-HNE), advanced glycation end products (AGE), advanced oxidation protein products (AOPP), dityrosine (DT), glutathione (GSH), kynurenine (KN), malondialdehyde (MDA), N-formyl kynurenine (NFK), total antioxidant capacity (TAC), total oxidative stress (TOS), tryptophan (TRY);
(c) Whole blood: selenium (Se), zinc (Zn) and arsenic (As).

##### 2.2.1.2 Laboratory analysis

The activity of CAT, GPx, SOD, GSH, AOPP, MDA, TAC, and TOS was determined colourimetrically. AGE, DT, KN, NFK, and TRY concentrations were determined fluorimetrically. 3-NT and 4-HNE concentrations were determined spectrophotometrically using the enzyme-linked immunosorbent assay (ELISA) method. All parameters were estimated in duplicate samples and standardized to 100 mg of total protein. The absorbance/fluorescence was measured using Infinite M200 PRO Multimode Microplate Reader, Tecan. The procedures were described in detail in our previous papers (Maciejczyk et al., 2019; Juchnowicz et al., 2021a,2021b,^2023^; Więdłocha et al., 2023).

The concentration of the trace elements: Se, Zn, and As, in whole blood, was measured by inductively coupled plasma mass spectroscopy (ICP-MS) technique using the Elan DRC-e instrument (PerkinElmer, USA). All the steps were performed according to standardized protocol to maximize precision and ensure repeatability of assessment. The determinations were performed in a laboratory member of the external quality control program (QMEQAS) of the Center du Toxicologie de Quebec. Methane was used to reduce polyatomic interference. Calibration standards were prepared by diluting the standard (Multi-Element Calibration Standard 3 10 mg/L; PerkinElmer Pure Plus, PerkinElmer Life and Analytical Sciences, USA) with 1% nitric acid (Suprapur^®^ HNO3; Merck, Germany). Separate calibration curves were prepared for each tested element using the following concentrations: 1, 10, 20, 50 and 100 μg/l. To compensate instrument drift and matrix effects, germanium (PerkinElmer Pure, PerkinElmer Life and Analytical Sciences, USA) was used as an internal standard.

#### 2.2.2 Sociodemographic and clinical assessment

A well-trained psychiatrist evaluated all individuals from the BD group to confirm the diagnosis. During the examination, information about the following socio-demographic and clinical variables was collected: age, gender, age of onset, duration of the disease, number of hospitalizations, presence and type of comorbidities, current pharmacological treatment and supplement taking, presence of comorbidities, body weight and height, tobacco smoking and psychoactive substance using, presence and type of family history for mental disorders and cancers. The same information, except the course of disease, was obtained from the HC group.

### 2.3 Statistical analysis

Statistica 13 software (TIBCO Software Inc., Palo Alto, CA, USA) was used for the analysis; results were considered statistically significant at *p* < 0.05. Firstly, the Shapiro–Wilk test was applied to determine the distribution of quantitative variables. Due to both ex-Gaussian and non-ex-Gaussian distribution, parametric and non-parametric tests were adopted. We used the Chi-squared test (categorical variables), Student’s t-test (ex-Gaussian distribution) and Man–Whitney U (non-ex-Gaussian distribution) to determine the differences between study groups. Correlation matrix (ex-Gaussian distribution) and Spearman’s rho correlation (ex-Gaussian distribution) were used to calculate the magnitude and direction of the relationship between quantitative variables.

#### 2.3.1 Data mining analysis

Data mining methods allow for uncovering valuable information from large data sets. We applied classification and regression tree (C&RT) analysis, frequently used to identify factors associated with outcomes of interest to the researcher. C&RT allows building a model capable of finding the most predictive biological factors from examined variables and its cut-off point related to BD. In the results, a multipanel of potential disease biomarkers was identified. It is also possible to identify biomedical variables related to another outcome (type of treatment, remission, etc.) in the C&RT method. In our model, biological variables (oxidative stress biomarkers and trace element concentration) were analyzed to determine prognostic factors of BD.

## 3 Results

### 3.1 Characteristic of examined population

The characteristics of the study population are shown in Table 1. We found no difference in gender, age, or BMI between examined groups. Most of the individuals were men and were in the fourth decade of life. The $\bar{\text{X}}$ of BMI of both groups were upon reference value. The BD group had more smokers compared to HC. The range of disease duration and number of hospitalizations in the BD group was wide. As has been shown in Table 2, one-third of patients suffered from chronic disease, the most often cardiovascular diseases, diabetes mellitus and thyroid diseases (*n* = 4).

**TABLE 1:** Characteristics of examined groups.

Examined factor	BD (*n* = 62)	HC (*n* = 40)	BD vs. HC
Gender, *N* (%) females	23 (37)	15 (38)	0.970
Age, Me (min–max)	37 (18–65)	40 (20–64)	0.450
BMI (kg/m^2^), X̄ (SD)	27.9 (5.1)	25.8 (4.2)	0.0640
Chronic disease, *N* (%)	21 (34)	6 (18)	0.220
Current smokers, *N* (%)	28 (45)	7 (15)	0.007*
Age of onset, Me (min–max)	24 (13–60)	N/A	
Duration of the disease (in months), Me (min–max)	84 (2–444)		
Number of hospitalizations, Me (min–max)	3 (0–19)		

BMI, body mass index; Me, median; X̄, mean; SD, standard deviation; min, minimum; max, maximum; BD, bipolar disorder; HC, healthy controls;

*significant difference between BD and HC groups; N/A, not applicable.

**TABLE 2:** Chronic diseases in patients group.

Chronic disease	BD; *N* (%)	HC; *N* (%)
Cardiovascular diseases	7 (11)	3 (8)
Diabetes mellitus	6 (8)	0 (0)
Thyroid diseases	4 (6)	0 (0)
Autoimmune diseases	2 (3)	0 (0)
Peripheral neuropathy	2 (3)	0 (0)
Lipid metabolism disorders	2 (3)	0 (0)
Chronic obstructive pulmonary disease	1 (2)	0 (0)
Atopic dermatitis	1 (2)	0 (0)
Gout	1 (2)	0 (0)
Gastroesophageal reflux disease	1 (2)	0 (0)
Asthma	1 (2)	2 (5)
Allergy	0 (0)	1 (3)
Irritable bowel syndrome	0 (0)	1 (3)
Seborrheic dermatitis	0 (0)	1 (3)

BD, bipolar disorder; HC, healthy controls.

### 3.2 Pro/anti-oxidant state

We found differences in 5 of 15 examined variables examined pro/anti-oxidant balance between analyzed groups (Table 3). The BD group had a higher concentration of AOPP, TAC, and CAT (*p* = 0.003; *p* = 0.04; *p* = 0.021, respectively) and a lower concentration of 4-HNE, GSH, and GPx (*p* < 0.001; *p* < 0.001; *p* = 0.034, respectively) compared to the HC group. Many differences in pro/anti-oxidant biomarkers (5 of 15 variables) were found between males and females, yet only in the HC group (data are available in Supplementary Table 1). However, it should be noted that the male and female groups differed in age (M > F; *p* = 0.041), which could affect the pro/anti-oxidant state.

**TABLE 3:** Differences in pro-/anti-oxidant state between examined group.

Examined factor	BD (*n* = 43) $\bar{\text{X}}$ ± SD/ Me (min–max)	HC (*n* = 30) $\bar{\text{X}}$ ± SD/ Me (min–max)	*p*-value
**Prooxidant biomarkers**			
3-NT (nmol/mg protein)	2.96 (1.45)	2.4 (1.27)	0.090
4-HNE (uM/mg protein)	0.003 (0.001–0.010)	0.006 (0.002–0.008)	< 0.001*
AGE (AFU/mg protein)	16.71 (11.26–26.42)	15.91 (10.15–27.42)	0.279
AOPP (umol/mg protein)	0.054 (0.023–0.14)	0.032 (0.018–0.108)	0.003*
DT (AFU/mg protein)	13.44 (7.41–26.84)	13 (7.04–20.55)	0.211
KN (AFU/mg protein)	19.86 (13.01–37.83)	21 (14.82–40.12)	0.228
MDA (umol/mg protein)	0.007 (0.002–0.01)	0.008 (0.003–0.0091)	0.110
NFK (AFU/mg protein)	15.47 (9.51–27.14)	14.32 (9.78–30.52)	0.159
**Antioxidant biomarkers**			
TAC (Trolox mmol/mg protein)	0.0017 (0.0003)	0.0016 (0.0003)	0.040*
TOS (umol H2O2 Equiv/mg protein)	0.002 (0.0002–0.048)	0.002 (0.0001–0.011)	0.239
TRY (AFU/mg protein)	296.96 (208.18–351.95)	298.14 (212.38–377.74)	0.644
GSH (ug/mg protein)	0.0109 (0.005)	0.0155 (0.005)	< 0.001*
CAT (umol H2O2/min/mg protein)	0.003 (0.001–0.012)	0.002 (0.001–0.005)	0.021*
GPx (U/mg protein)	0.406 (0.147–0.886)	0.628 (0.281–0.844)	0.034*
SOD (U/mg protein)	0.021 (0.006–0.082)	0.034 (0.009–0.082)	0.206

$\bar{\text{X}}$, mean; SD, standard deviation; Me, median; min, minimum; max, maximum; BD, bipolar disorder; HC, healthy controls; 3-NT, 3-nitrotyrosine, 4-HNE, 4-hydroxynonenal; AGE, advanced glycation end products; AOPP, advanced oxidation protein products; DT, dityrosine; KN, kynurenine; MDA, malondialdehyde; NFK, N-formyl kynurenine; TAC, total antioxidant capacity; TOS, total oxidative stress; TRY, tryptophan; GSH, glutathione; CAT, catalase; GPx, glutathione peroxidase; SOD, superoxide dismutase;

*significant difference between BD and HC groups.

The relationship between pro/anti-oxidant biomarkers and socio-demographic, clinical data has been shown in Supplementary Tables 2, 3. From variables significantly differentiated examined group, age was positively associated with AOPP (*R* = 0.57, *p* = 0.001), CAT (*R* = 0.63, *p* < 0.001) and negatively with TAC (*R* = −0.47, *p* = 0.008), GPx (*R* = −0.5, *p* = 0.005) in HC group. In the BD group, only a correlation between age and GHS was found (*R* = −0.37, *p* = 0.043). Correlations were also found between variables significantly differentiating the examined group and BMI. In the HC group, positive with AOPP (*R* = 0.53, *p* = 0.004) and CAT (0.4, *p* = 0.020); in the BD group, positive with CAT (*R* = 0.41, *p* = 0.033) and negative with GPx (*R* = −0.44, *p* = 0.010). Some weak correlations between the pro/anti-oxidant state and the variables related to the course of the disease of the BD group were found, as shown in Table 4.

**TABLE 4:** Relationship between concentration of oxidative stress biomarkers and the course of the disease.

Examined relationship	R Spearman	*p-value*
4-HNE & Number of hospitalizations	0.37	0.040*
DT & Number of hospitalizations	0.36	0.044*
MDA & Number of hospitalizations	−0.44	0.012*
NFK & Number of hospitalizations	0.37	0.036*
AGE & DD of Li	−0.46	0.008*
NFK & DD of Li	−0.50	0.004*
3-NT & DD of VA	0.36	0.047*
NFK & DD of VA	0.42	0.017*
TAC & DD of VA	−0.45	0.011*
TRY & DD of VA	−0.39	0.034*
CAT & DD of VA	0.41	0.027*
GPx & DD of VA	−0.52	0.004*

4-HNE, 4-hydroxynonenal; DT, dityrosine; MDA, malondialdehyde; NFK, N-formyl kynurenine; 3-NT, 3-nitrotyrosine; TAC, total antioxidant capacity; TRY, tryptophan; CAT, catalase; GPx, glutathione peroxidase; Li, lithium; VA, valproic acid; DD, daily dose;

*significant relationship.

### 3.3 Trace elements

Table 5 depicts the concentration of trace elements in the whole blood of the examined population. The BD group represented the lower concentration of all examined trace elements than the HC group. In the HC group, all trace element levels were lower in females than males, while the females and males from the BD group were not differentiated with trace element concentration in the blood.

**TABLE 5:** Differences in trace element blood concentration between the examined groups.

Examined factor	BD (*n* = 62)	HC (*n* = 40)	*p*-value
Se (μg/l), Me (min–max)	95.33 (60.82–192.53)	110.94 (68.30–146.23)	0.002*
Zn (μg/l), X (SD)	6,186.85 (850.34)	6,604.85 (722.60)	0.014*
As (μg/l), Me (min–max)	0.80 (0.16–5.03)	1.12 (0.35–6.62)	0.002*

BD, bipolar disorder; HC, healthy controls; X, mean; SD, standard deviation; Me, median; min, minimum; max, maximum; Se, selenium; Zn, zinc; As, arsenic;

*significant difference between BD and HC groups.

We found a positive relationship between Zn concentration and BMI in the HC group (*R* = 0.46, *p* = 0.009) and a negative between As and BMI (*R* = −0.41, *p* = 0.006) (see Supplementary Table 4). In individuals from the BD group who smoked cigarettes, the inverse relationship between Se concentration and number of cigarettes smoked per day was found (*R* = −0.34, *p* = 0.020). Lower Se concentration was related to the duration of illness in the BD group (*R* = −0.34, *p* = 0.037) (see Supplementary Table 5). Se concentration was positively associated with Zn concentration in the HC group (*R* = 0.36, *p* = 0.022) and in both groups with As (BD: *R* = 0.42, *p* = 0.008; HC: *R* = 0.45, *p* = 0.005) (see Supplementary Table 6).

Trace minerals concentration was related to some markers of oxidations (Table 6). In the BD group, mainly inversely–Se with AGE; DT and KN with NFK, while As was positively correlated with NFK. In the HC group, positively correlations were found between Se and AGE, Se and NFK and Zn with 3-NT. Regarding the relationship between pro/anti-oxidants and trace elements, in the BD group, Se was inversely associated with TAC. In the HC group, Se was positively associated with CAT, Zn negatively with TAC and positively with CAT. We did not find any relationship between Li or VA treatment and concentration of trace minerals (differences between groups or relationship with dose of medication).

**TABLE 6:** Relationship between trace elements and pro-/antioxidant state biomarkers in the blood of examined groups.

Examined relationship	Group	R Spearman	*p-value*
Se & AGE	BD	−0.32	0.039*
	HC	0.37	0.040*
Se & DT	BD	−0.50	< 0.001*
	HC	0.17	0.356
Se & KN	BD	−0.38	0.015*
	HC	0.22	0.251
Se & NFK	BD	−0.13	0.424
	HC	0.38	0.038*
Se & TAC	BD	−0.33	0.031*
	HC	−0.65	0.732
Se & TRY	BD	−0.39	0.012*
	HC	−0.01	0.950
Se & CAT	BD	0.14	0.365
	HC	0.38	0.040*
Zn & 3-NT	BD	0.27	0.079
	HC	0.43	0.019*
Zn & TAC	BD	−0.15	0.352
	HC	−0.43	0.017*
Zn & CAT	BD	0.14	0.389
	HC	0.50	0.006*
As & NFK	BD	0.44	0.004*
	HC	0.29	0.122

BD, bipolar disorder; HC, healthy controls; Se, selenium; Zn, zinc; As, arsenic; AGE, advanced glycation end products; DT, dityrosine; KN, kynurenine; NFK, N-formyl kynurenine; TAC, total antioxidant capacity; TRY, tryptophan; CAT, catalase;

*significant relationship.

### 3.4 Biological predictors of bipolar disorder

**FIGURE 1:**
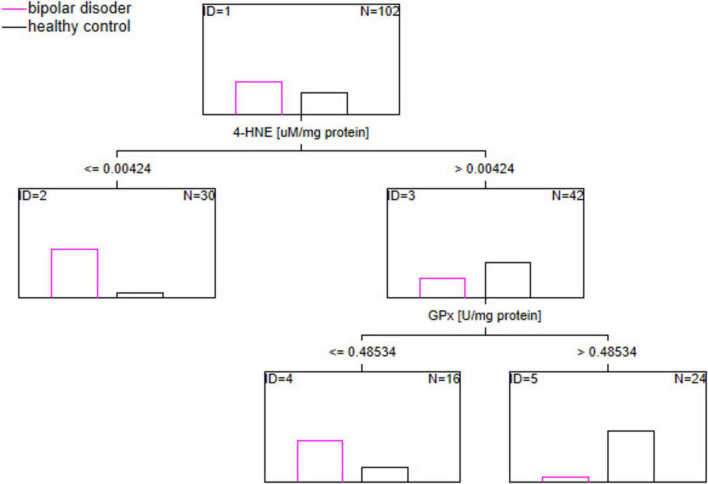
The potential multipanel of biological markers related to BD. *N*, number of analyzed blood samples; ID, identification number of nodes which sort down data.

C&RT analysis was applied to find the potential multipanel of biological markers related to BD. The results are depicted in Figure 1. According to the analysis, the most useful factors for discriminating the BD group from the HC group were 4-HNE (cut-off: ≤ 0.004 uM/mg protein) and GPx (cut-off: ≤ 0.485 U/mg protein). The proposed model allocated examined population in BD and HC groups with 90.5% accuracy, according to the area under receiver operating characteristic (ROC) curve (AUC).

## 4 Discussion

The study aimed to identify the potential changes related to pro/anti-oxidant balance in BD patients and their relationship with some trace elements and assess the potential utility of the OS biomarkers in classifying the patients as diagnosed with BD. OS biomarkers: total antioxidant capacity (TAC), catalase (CAS), and advanced oxidation protein products (AOPP) were higher in the BD group compared to HC, whereas concentration of 4-hydroxynonenal (4-HNE), glutathione (GSH), glutathione peroxidase (GPx) was lower in BD compared to HC. We found lower concentrations of all examined trace elements (selenium, zinc and arsenium) in the BD group compared to HC.

Other studies in treated and drug-free patients confirmed the relationship between higher concentrations of advanced oxidation protein products (AOPP) and BD (Maes et al., 2019; Guidara et al., 2022, 2023). The free radicals and other oxidizing compounds lead to the oxidation of proteins and pathological changes in protein structures (Selmeci, 2011). The consequences of these processes are changes in enzyme activity (also observed in our study), changes in protein function, and their aggregation or proteolysis. According to Maes, protein oxidation with the formation of the AOPP is characteristic of BDI but not BDII patients. Biological differences between BDI and BDII remain a relatively obscure field. Most examinations have not compared patients based on the type of BD to date (Andreazza et al., 2008; Brown et al., 2014). AOPP is supposed to be involved in the severity of depressive symptoms (Maes et al., 2019). Maes also found a higher concentration of AOPP in males, which was not confirmed by our study. In the study which compared AOPP concentration between BD, schizophrenia patients and healthy individuals, only the schizophrenia acute psychotic phase was related to AOPP. However, a small sample size (*N* = 18 in each group) should be accounted (Tunçel et al., 2015). AOPP is a marker that measures highly oxidized proteins such as albumin. The role of AOPP in inflammation was found via the RAGE-NF-κB pathway (Lou et al., 2021). In our study (similar to Maes’s), AOPP was related to age and BMI, but we found this connection only in healthy individuals (Maes et al., 2019).

Total antioxidant capacity (TAC) allows the estimate of the extra-cellular non-enzymatic antioxidants by different (direct or indirect methods) (Silvestrini et al., 2023). One study found higher TAC in the BD group than in healthy individuals; however, the results did not reach statistical significance. The study’s lack of association between TAC and BD could result from the euthymic state of the examined patient group (Tozoglu et al., 2021). The authors showed a positive relationship between TAC and executive functioning. Akarsu et al. (2018) showed no difference in TAC between patients with first-episode mania and those with more than one episode of mania with higher levels in both groups compared to healthy individuals. These findings suggest that increased antioxidant mechanisms by exposure to oxidative stress are independent of the number of manic episodes or disease duration. In our study, TAC concentration in the BD group was found to be higher than HC, even though VA was inversely related to TAC concentration.

In our study, catalase (CAT) concentration in patients’ blood was higher compared to healthy individuals. CAT acts as an antioxidant by removing excess intracellular hydrogen peroxide and reactive oxygen species. In the mouse model, the excess brain intracellular hydrogen peroxide led to CAT inactivation and, through dysfunction of the serotonergic system, induced depressive behavior (Xu et al., 2024). According to meta-analyses conducted in 2008, 2014 and 2022, CAT was not related to BD (Andreazza et al., 2008; Brown et al., 2014; Jiménez-Fernández et al., 2022). However, a meta-analysis of 8 studies from 2020 found higher CAT in BD patients compared to the healthy group, and CAT levels did not significantly differ in patients with mania, depression, or euthymia. CAT is an enzyme that works in acute situations when oxidative stress appears and some antidepressants have been shown to affect ACT activity (Jiménez-Fernández et al., 2022; Xu et al., 2024).

The most attention should be paid to 4-hydroxynonenal (4-HNE) in patients with BD. In our study, the marker had a lower concentration in the patient group and was positively related to the number of hospitalizations. Moreover, data mining analysis proposed 4-HNE as a potential biomarker of BD with a cut-off point ≤ 0.004 uM/mg protein. Surprisingly, lower 4-HNE was related to the disease. 4-HNE is involved in lipid metabolism and processes linked with mitochondrial and antioxidant effects. 4-HNE is produced during lipid peroxidation (unsaturated fatty acids) in cells and is recognized as a lipid marker of this process (Scola et al., 2016). The studies confirm robust deficits of blood unsaturated fatty acids in BD, which could explain the obtained results to some extent (McNamara and Welge, 2016; Saito et al., 2023). A non-significant increase of 4-HNE and their relation with depression symptoms was shown in individuals with a high risk for BD, but in older patients (mean age 63.9), the differences in 4-HNE were not found (Andreazza et al., 2015; Scola et al., 2016). According to a post-mortem study, 4-HNE levels were higher in BD patients’ anterior cingulate brains than in healthy individuals. They increased by 94% in medication-free BD patients compared to medicated patients (Wang et al., 2009). The model study found Li treatment prevents 4-HNE-protein adduction in the rat frontal cortex (Tan et al., 2012). In our examination, 4-HNE was not related to VA or Li treatment.

According to our analysis, glutathione (GSH) concentration was lower in the BD group than in healthy individuals and lower in older individuals (only in the patient’s group). Some relation of GSH with oxidative damage (AOPP, 3-NT) was also found in BD. These results are consistent with a meta-analysis of 8 studies (Jiménez-Fernández et al., 2021). Stratification by different illness phases/polarity showed lower GSH only in euthymia and without differences in patients with mania compared to healthy individuals. Some authors proposed GSH as a trait marker of BD (Jiménez-Fernández et al., 2022). However, our data-mining analysis did not confirm this suggestion.

The glutathione peroxidase (GPx) is a family of selenoproteins with highly various structures and functions. With cooperation with GSH, it is potent to hydrogen peroxide (Steinbrenner et al., 2016). In our study, GPx and GSH, concentrations were lower in patients with BD compared to healthy individuals. We observed an inverse relationship between GPx and age, but only in the HC group. A lower concentration of GPx was related to higher BMI in the patients group. Our study suggests VA could have a negative impact on GPx concentration. In patients with BD, a negative correlation between GPx and brain-derived neurotrophic factor (BDNF) was shown, and the higher GPx was explained as a compensatory mechanism (Mansur et al., 2016). Nevertheless, a meta-analysis of 11 studies conducted in 2020 did not confirm differences between healthy subjects and patients regarding the activity of the GPx (Jiménez-Fernández et al., 2022). However, authors showed patients during mania episodes and without treatment had a significantly lower GPx activity compared to healthy individuals. GPx was lower in patients during depression episodes in one study, and six weeks of Li therapy did not affect these changes (de Sousa et al., 2014). GPx regularly scavenges free radicals that are constantly formed in the organism, and lower concentrations in BD patients could result from the exhaustion of these supplies.

In our analysis, the examined groups differed by all examined trace elements (selenium–Se, zinc–Zn, arsenic–As), and lower concentration was observed in the patient groups (Chowdhury et al., 2017; Millett et al., 2017; Santa Cruz et al., 2020; Chebieb et al., 2023).

It has been shown the level of zinc (Zn) was decreased in patients with mania or hypomania with correlation to the number of manic relapses in the past year and during remission was similar to the level of healthy individuals (Siwek et al., 2016). The level of Zn was also related to depression severity in women who had BD (Millett et al., 2017). Surprisingly, in two studies, serum Zn levels were higher in patients with BD than in healthy individuals (González-Estecha et al., 2011; Jonsson et al., 2022). Nevertheless, these results have no clinical implications, such as correlation with pro/anti-inflammatory markers, executive functioning or disease severity (Jonsson et al., 2022). Authors observed higher concentrations of Zn in patients who declared cannabis and cocaine consumption, which was confirmed by other studies with healthy individuals (González-Estecha et al., 2011). Limited evidence suggests no changes in Zn concentration after treatment of BD and no differences between the concentration of Zn between patients with BD and those with depression (Styczeń et al., 2017; Sampath et al., 2022).

Zn acts as a modulator of neurotransmission in the brain, allowing a balance between the glutamatergic and GABA-ergic systems. Zn affects BDNF synthesis, and their supplementation in patients with depression increases serum BDNF levels. Zn also has an antioxidant action (Mousavi et al., 2020). In our study, their concentration was related to markers of oxidative stress: inversely with TAC and positively with 3-NT and catalase, but only in healthy subjects. Contrary to our results, according to meta-analysis, Zn supplementation positively affected TAC (Mousavi et al., 2020). In the study of children with neuropathic bladder, CAT was inversely associated with Zn concentration (Nabieh et al., 2023).

Siwek et al. (2013) suggested Zn as a clinical state or trait marker of affective disorders. Despite this proposal, more than a decade has passed, and the role of Zn in affective disorder remains unclear.

The different results may be an effect of the assessment of various fractions of blood. Usually, circulating trace elements are not uniformly distributed between plasma and cellular blood components; most are found in white or red blood cells (RBCs). Concentrations of trace elements in RBCs most often reflect the intracellular stores and general homeostasis (Skalnaya and Skalny, 2018).

Studies on the animal models suggested the connection between Li therapy and selenium (Se) status, but our study did not confirm these results (Kiełczykowska et al., 2017, 2020; Soliman et al., 2023). A lower concentration of Se serum was found in BD patients compared to healthy individuals. The animal model showed a protective effect of Se supplementation against Li-induced thyroid toxicity. Considering the harmful effects on thyroid structure and function during long-term therapy of Li and, proven by us, lower levels of Se in the blood of patients with BD, and the inverse relationship between Se and the disease duration, the clinical utility of Se supplementation should be tested in further investigation (Soliman et al., 2023). In our study, the higher Se concentration also implicated lower levels of OS biomarkers in the patients group: AGE, DT, KN and also TAC, TRY. These correlations suggest the role of Se not only in oxidative mechanisms but also in the modulation of the kynurenine pathway (KN, DT, TRY) in BD.

According to one study, patients treated with Li had lower serum Se, but the sample size was relatively small (7 patients on Li therapy and 8 treated with other medication) (Santa Cruz et al., 2020). Li is supposed to have an antagonist effect for Se in BD patients. Nevertheless, our study did not confirm these results, and this issue needs further investigation, especially in human studies. We did not confirm a relationship between Se concentration and VA treatment in the study group. Little evidence from model studies suggests Zn and Se supplementation protects against liver damage induced by VA (Ahangar et al., 2017). We did not examine the liver function; these promising findings need clarification.

Arsenic (As) is one of the three most toxic heavy metals, according to the US Agency for Toxic Substances and Disease Registry (ASTDR, 2008). It has been shown As affects adult neurogenesis, hippocampus-dependent learning and memory. A dose-dependent relationship between cognitive impairment, BDNF concentration and As exposure in humans has been shown (Karim et al., 2019). We examined the total concentration of As. It should be noted this trace element exists in several forms, both toxic (As 5+, As3+) and non-toxic. As become integrated into nonvascular tissues up to 2 days after exposure, urine concentration is the proposed marker to assess As exposure (Mayo Clinic Laboratories, 2024). However, plasma blood samples are suggested to be the best indicator of low-level exposure to As. In one study, whole-blood As levels were associated with a higher risk of OS (Tan et al., 2021). The group we examined had low exposure to As, and lower concentration was found in the BD group compared to healthy individuals. These findings could be partially explained by a higher concentration of Se in the healthy group. According to earlier findings, the high rate of As urine washout was significantly associated with the high total selenium urine excretion (Janasik et al., 2017). In our study, As levels in the BD group were positively related to toxic NFK. Hypothetically, As urine washing out in the patients’ group could be a mechanism against oxidative stress and affect the observed lower concentration of As in blood samples.

The OS changes, and trace element concentrations are highly affected by dietary patterns not examined in this study. The peripheral concentration may not reflect the level of OS markers in the cerebrospinal fluid and nutritional status/exposure of trace elements (Pasquali et al., 2015; Gigase et al., 2023). Further studies should assess the combination of peripheral levels from various fluids. However, only a few studies have examined dietary intake in BD, and this issue needs more comprehensive studies (Jacka et al., 2011; Bly et al., 2014; Teasdale et al., 2019).

## 5 Strengths and limitations of the study

To the best of our knowledge, this is the first study to assess the OS and trace elements of blood concentration changes in BD patients and their relationship with treatment. However, some of the limitations must be mentioned.

The cross-sectional character of the study makes it impossible to evaluate the cause-effect relationships. The examined group of patients was relatively small, and we did not assess the relationship between biological factors and the severity of symptoms among them. Further studies should consider the number of BD episodes and the disease phase. A sub-group analysis based on the type of BD needed to be included also. The examined group was treated with medication other than Li and VA. The interaction between drugs could affect the examined biological factors (Bhattacharyya et al., 2014). We measured the levels of OS biomarkers in plasma and serum and trace elements in whole blood. Some individuals from the BD group also had chronic conditions that affected the examined markers. However, the strict exclusion criteria were intended to minimize the effect of multimorbidity. The diversity of symptoms, stage of BD might have affected the results. Smoking also has a significant impact on OS, and the compared group significantly differed in terms of smoking. More information about smoking patterns should be collected and analyzed with biological data in the future.

## 6 Conclusion

The above results may indicate pro/antioxidant state disturbances and changes in trace elements blood concentration in patients who have bipolar disorder. Higher levels of OS and lower levels of blood trace elements concentration in BD may, at least partially, play a role in the pathophysiology of BD and be a potential target in further pharmacotherapeutic interventions. Some OS-related biological indicators from blood may be used as a multipanel of BD biomarkers. The pro/antioxidant state is related to the Li (in favor of antioxidant properties of Lithium) and VA (in favor of prooxidant properties of Valproic Acid) treatment.

## Data availability statement

The raw data supporting the conclusions of this article will be made available by the authors, without undue reservation.

## Ethics statement

The studies involving humans were approved by the Ethics Committee of the Medical University of Lublin, Lublin, Poland. The studies were conducted in accordance with the local legislation and institutional requirements. The participants provided their written informed consent to participate in this study.

## Author contributions

JR: Conceptualization, Data curation, Formal analysis, Investigation, Methodology, Visualization, Writing – original draft, Writing – review & editing. ŁŁ: Conceptualization, Data curation, Investigation, Methodology, Writing – review & editing. MH: Data curation, Investigation, Writing – review & editing. WM: Formal analysis, Investigation, Writing – review & editing. RD: Formal analysis, Investigation, Writing – review & editing. AK: Formal analysis, Investigation, Writing – review & editing. MiM: Formal analysis, Investigation, Writing – review & editing. JL: Formal analysis, Funding acquisition, Investigation, Methodology, Supervision, Writing – review & editing. MN: Formal analysis, Investigation, Writing – review & editing. MŻ-P: Formal analysis, Investigation, Writing – review & editing. AZ: Formal analysis, Investigation, Writing – review & editing. MaM: Formal analysis, Investigation, Methodology, Supervision, Writing – review & editing. HK-J: Conceptualization, Funding acquisition, Methodology, Supervision, Writing – original draft, Writing – review & editing.
