# Supplementary material for: Pro/antioxidant status and selenium, zinc and arsenic concentration in patients with bipolar disorder treated with lithium and valproic acid
Source: Front Mol Neurosci. 2024 Sep 11;17:1441575. doi: 10.3389/fnmol.2024.1441575 (PMC11423611; doi:10.3389/fnmol.2024.1441575)
Supplement: Supplementary file 1 [file Table_1.docx]

**Table S1. Differences in blood pro-/antioxidant state biomarkers dependent on gender**

| **Examined factor** | \| **Group** \| \| --- \| | \| **Gender** \| \| --- \| | **Me (Min-Max)** | \| ***p-value*** \| \| --- \| |
| --- | --- | --- | --- | --- | --- | --- | --- |
| 3-NT | HC | F | 1.13 (0.71-2.44) | **0.011*** |
|  |  | M | 2.64 (0.71-5.43) |  |
|  | BD | F | 1.81 (0.98-4.88) | 0.063 |
|  |  | M | 3.09 (0.89-6.39) |  |
| AOPP | HC | F | 0.02 (0.02-0.04) | **0.012*** |
|  |  | M | 0.04 (0.02-0.11) |  |
|  | BD | F | 0.04 (0.02-0.11) | 0.056 |
|  |  | M | 0.06 (0.02-0.14) |  |
| TAC | HC | F | 0.0017 (0.0012-0.0024) | **0.018*** |
|  |  | M | 0.0015 (0.0008-0.0019) |  |
|  | BD | F | 0.0018 (0.0013-0.0025) | 0.143 |
|  |  | M | 0.0017 (0.0011-0.0024) |  |
| CAT | HC | F | 0.0017 (0.0014-0.0028) | **0.022*** |
|  |  | M | 0.0021 (0.0016-0.0047) |  |
|  | BD | F | 0.0025 (0.0014-0.0115) | 0.252 |
|  |  | M | 0.0035 (0.0016-0.0102) |  |
| GPx | HC | F | 0.71 (0.50-0.84) | **0.032*** |
|  |  | M | 0.61 (0.28-0.82) |  |
|  | BD | F | 0.50 (0.15-0.89) | 0.262 |
|  |  | M | 0.39 (0.15-0.85) |  |

F – females; M – males; X – mean; SD – standard deviation; Me – median; min – minimum; max – maximum; BD – bipolar disorder; HC – healthy controls; 3-NT - 3-nitrotyrosine; AOPP - advanced oxidation protein products; TAC - total antioxidant capacity; CAT – catalase; GPx - glutathione peroxidase; *significant difference between females and males groups

**Table S2. Relationship between pro/antioxidant status and sociodemograpic data of examined groups**

| **Examined relationship** | \| **Group** \| \| --- \| | \| **R Spearman** \| \| --- \| | \| ***p-value*** \| \| --- \| |
| --- | --- | --- | --- | --- | --- | --- |
| 3-NT & Age | BD | 0.09 | 0.617 |
|  | HC | **0.5** | **0.005*** |
| AOPP & Age | BD | 0.19 | 0.263 |
|  | HC | **0.57** | **0.001*** |
| MDA & Age | BD | -0.14 | 0.397 |
|  | HC | **-0.51** | **0.004*** |
| TAC & Age | BD | -0.01 | 0.964 |
|  | HC | **-0.47** | **0.008*** |
| TRY & Age | BD | -0.13 | 0.447 |
|  | HC | **-0.44** | **0.038*** |
| CAT & Age | BD | 0.25 | 0.141 |
|  | HC | **0.63** | **<0.001*** |
| GPx & Age | BD | -0.25 | 0.142 |
|  | HC | **-0.5** | **0.005*** |
| GSH & Age | BD | **-0.37** | **0.043*** |
|  | HC | 0.17 | 0.245 |
| 3-NT & BMI | BD | 0.1 | 0.583 |
|  | HC | **0.58** | **0.001*** |
| AGE & BMI | BD | 0.07 | 0.717 |
|  | HC | **0.38** | **0.043*** |
| AOPP & BMI | BD | 0.11 | 0.537 |
|  | HC | **0.53** | **0.004*** |
| MDA & BMI | BD | -0.09 | 0.636 |
|  | HC | **-0.47** | **0.012*** |
| CAT & BMI | BD | **0.4** | **0.02*** |
|  | HC | **0.41** | **0.033*** |
| GPx & BMI | BD | **-0.44** | **0.01*** |
|  | HC | -0.26 | 0.183 |

BD – bipolar disorder; HC – healthy controls; BMI – body mass intex; 3-NT - 3-nitrotyrosine; AOPP - advanced oxidation protein products; MDA – malondialdehyde; TAC - total antioxidant capacity; TRY – tryptophan; GPx - glutathione peroxidase; GSH – glutathione; CAT – catalase; *significant difference between BD and HC groups

**Table S3. Relationship between concentration of blood pro-/antioxidant state biomarkers of examined groups (only correlation R<0.50 were shown and regarding as statistically significant)**

| **Examined relationship** | \| **Group** \| \| --- \| | \| **R Spearman** \| \| --- \| | \| ***p-value*** \| \| --- \| |
| --- | --- | --- | --- | --- | --- | --- |
| \| 3-NT & AOPP \| \| --- \| | BD | **0.92** | **<0.001*** |
|  | HC | **0.89** | **<0.001*** |
| \| 3-NT & DT \| \| --- \| | BD | -0.44 | 0.003 |
|  | HC | **-0.67** | **<0.001*** |
| \| 3-NT & GSH \| \| --- \| | BD | **0.54** | **<0.001*** |
|  | HC | 0.40 | 0.031 |
| \| 3-NT & TAC \| \| --- \| | BD | -0.35 | 0.022 |
|  | HC | **-0.74** | **<0.001*** |
| \| 3-NT & CAT \| \| --- \| | BD | **0.67** | **<0.001*** |
|  | HC | 0.39 | 0.034 |
| \| 3-NT & PX \| \| --- \| | BD | **-0.67** | **<0.001*** |
|  | HC | -0.34 | 0.069 |
| \| AGE & DT \| \| --- \| | BD | **0.80** | **<0.001*** |
|  | HC | **0.57** | **0.001*** |
| \| AGE & KN \| \| --- \| | BD | **0.75** | **<0.001*** |
|  | HC | **0.77** | **<0.001*** |
| \| AGE & NFK \| \| --- \| | BD | **0.85** | **<0.001*** |
|  | HC | **0.87** | **<0.001*** |
| \| AGE & TRY \| \| --- \| | BD | **0.60** | **<0.001*** |
|  | HC | 0.39 | 0.067 |
| \| AOPP & DT \| \| --- \| | BD | -0.26 | 0.096 |
|  | HC | **-0.68** | **<0.001*** |
| \| AOPP & GSH \| \| --- \| | BD | **0.59** | **<0.001*** |
|  | HC | 0.35 | 0.060 |
| \| AOPP & TAC \| \| --- \| | BD | -0.11 | 0.495 |
|  | HC | **-0.63** | **<0.001*** |
| \| AOPP & TOS \| \| --- \| | BD | **-0.53** | **<0.001*** |
|  | HC | -0.21 | 0.265 |
| \| AOPP & CAT \| \| --- \| | BD | **0.78** | **<0.001*** |
|  | HC | **0.54** | **0.003*** |
| \| AOPP & PX \| \| --- \| | BD | **-0.77** | **<0.001*** |
|  | HC | **-0.54** | **0.003*** |
| \| DT & KN \| \| --- \| | BD | **0.71** | **<0.001*** |
|  | HC | 0.27 | 0.151 |
| \| DT & NFK \| \| --- \| | BD | **0.53** | **<0.001*** |
|  | HC | 0.24 | 0.198 |
| \| DT & TAC \| \| --- \| | BD | **0.63** | **<0.001*** |
|  | HC | **0.76** | **<0.001*** |
| \| DT & TRY \| \| --- \| | BD | **0.61** | **<0.001*** |
|  | HC | **0.59** | **0.003*** |
| \| KN & NFK \| \| --- \| | BD | **0.68** | **<0.001*** |
|  | HC | **0.85** | **<0.001*** |
| \| KN & TRY \| \| --- \| | BD | **0.53** | **<0.001*** |
|  | HC | 0.30 | 0.162 |
| \| GSH & 3-NT \| \| --- \| | BD | **0.54** | **<0.001*** |
|  | HC | 0.40 | 0.031 |
| \| GSH & TOS \| \| --- \| | BD | -0.45 | 0.002 |
|  | HC | 0.14 | 0.473 |
| \| TAC & TRY \| \| --- \| | BD | **0.53** | **<0.001*** |
|  | HC | 0.27 | 0.208 |
| \| TRY & PX \| \| --- \| | HC | **0.52** | **0.013*** |
|  | BD | 0.18 | 0.269 |
| \| CAT & PX \| \| --- \| | BD | **-0.99** | **<0.001*** |
|  | HC | **-0.91** | **<0.001*** |

3-NT - 3-nitrotyrosine; AGE - advanced glycation end products; AOPP - advanced oxidation protein products; DT – dityrosine; GSH – glutathione; KN – kynurenine; MDA – malondialdehyde; NFK - N-formyl kynurenine; TAC - total antioxidant capacity; TOS - total oxidative stress; TRY – tryptophan; CAT – catalase; GPx - glutathione peroxidase; SOD – superoxide dismutase; *significant relationship

**Table S4. Relationship between concentration of blood traces element and sociodemograpic data of examined groups**

| **Examined relationship** | \| **Group** \| \| --- \| | \| **R Spearman** \| \| --- \| | \| ***p-value*** \| \| --- \| |
| --- | --- | --- | --- | --- | --- | --- |
| Se & Age   \|  \| \| --- \| | BD | -0.19 | 0.168 |
|  | HC | 0.21 | 0.206 |
| Se & BMI | BD | -0.18 | 0.240 |
|  | HC | 0.32 | 0.086 |
| Zn & Age | BD | 0.02 | 0.872 |
|  | HC | 0.31 | 0.061 |
| Zn & BMI | BD | 0.12 | 0.431 |
|  | HC | **0.46** | **0.009*** |
| As & Age | BD | -0.18 | 0.190 |
|  | HC | 0.09 | 0.593 |
| As & BMI | BD | **-0.41** | **0.006*** |
|  | HC | 0.19 | 0.323 |

BD – bipolar disorder; HC – healthy controls; BMI – body mass index; Se – selenium; Zn – zinc; As – arsenic; *significant relationship

**Table S5. Relationship between concentration of blood traces elements and the clinical characteristic of BD group**

| **Examined relationship** | \| **R Spearman** \| \| --- \| | \| ***p-value*** \| \| --- \| |
| --- | --- | --- | --- | --- |
| Se & Age of onset | 0.15 | 0.307 |
| Zn & Age of onset | 0.04 | 0.778 |
| As & Age of onset | -0.09 | 0.568 |
| Se & Duration of the disease | **-0.34** | **0.037*** |
| Zn & Duration of the disease | 0.15 | 0.377 |
| As & Duration of the disease | -0.32 | 0.058 |
| Se & Number of hospitalization | -0.14 | 0.344 |
| Zn & Number of hospitalization | 0.07 | 0.651 |
| As & Number of hospitalization | -0.01 | 0.944 |

Se – selenium; Zn – zinc; As – arsenic; *significant relationship

**Table S6. Relationship between concentration of blood traces element of examined groups**

| **Trace elements** | \| **Group** \| \| --- \| | \| **R Spearman** \| \| --- \| | \| ***p-value*** \| \| --- \| |
| --- | --- | --- | --- | --- | --- | --- |
| \| Se & Zn \| \| --- \| | BD | 0.15 | 0.262 |
|  | HC | **0.36*** | **0.022*** |
| \| Se & As \| \| --- \| | BD | **0.42*** | **0.008*** |
|  | HC | **0.45*** | **0.005*** |
| \| Zn & As \| \| --- \| | BD | -0.02 | 0.901 |
|  | HC | 0.26 | 0.107 |

BD – bipolar disorder; HC – healthy controls; Se – selenium; Zn – zinc; As – arsenic; *significant relationship
